# Structural Insights into the Mechanisms of Action of Short-Peptide HIV-1 Fusion Inhibitors Targeting the Gp41 Pocket

**DOI:** 10.3389/fcimb.2018.00051

**Published:** 2018-02-26

**Authors:** Xiujuan Zhang, Yuanmei Zhu, Hao Hu, Senyan Zhang, Pengfei Wang, Huihui Chong, Jinsheng He, Xinquan Wang, Yuxian He

**Affiliations:** ^1^College of Life Sciences and Bioengineering, School of Science, Beijing Jiaotong University, Beijing, China; ^2^Institute of Pathogen Biology and Center for AIDS Research, Chinese Academy of Medical Sciences and Peking Union Medical College, Beijing, China; ^3^Ministry of Education Key Laboratory of Protein Science, Beijing Advanced Innovation Center for Structural Biology, School of Life Sciences, Tsinghua University, Beijing, China

**Keywords:** HIV-1, fusion inhibitor, six-helix bundle, crystal structure, resistance

## Abstract

The deep hydrophobic pocket of HIV-1 gp41 has been considered a drug target, but short-peptides targeting this site usually lack potent antiviral activity. By applying the M-T hook structure, we previously generated highly potent short-peptide fusion inhibitors that specifically targeted the pocket site, such as MT-SC22EK, HP23L, and LP-11. Here, the crystal structures of HP23L and LP-11 bound to the target mimic peptide N36 demonstrated the critical intrahelical and interhelical interactions, especially verifying that the hook-like conformation was finely adopted while the methionine residue was replaced by the oxidation-less prone residue leucine, and that addition of an extra glutamic acid significantly enhanced the binding and inhibitory activities. The structure of HP23L bound to N36 with two mutations (E49K and L57R) revealed the critical residues and motifs mediating drug resistance and provided new insights into the mechanism of action of inhibitors. Therefore, the present data help our understanding for the structure-activity relationship (SAR) of HIV-1 fusion inhibitors and facilitate the development of novel antiviral drugs.

## Introduction

Entry of HIV-1 into target cells involves the binding of the trimeric viral envelope glycoprotein (Env), which comprises the surface subunit gp120 and the transmembrane subunit gp41, to the cell receptor CD4 and chemokine coreceptor CCR5 or CXCR4, which triggers a barrage of conformational changes in Env complexes that activate the activity of gp41 (Chan et al., 1997; Eckert and Kim, 2001; Colman and Lawrence, 2003). In brief, dissociation of the gp120 from gp41 allows the N-terminal fusion peptide of gp41 to be exposed and inserted into the target cell membrane, resulting in the gp41 ectodomain to form a pre-hairpin configuration that bridges the virus and targeting cells. Then, three C-terminal heptad repeats (CHR) fold antiparalelly onto the trimeric coiled coil of the N-terminal heptad repeats (NHR) to adopt a thermostable six-helix bundle (6-HB) structure, which drives the viral and cellular membranes merger eventually leading to the occurrence of fusion (Chan et al., 1997; Tan et al., 1997; Weissenhorn et al., 1997).

**Figure 1:**
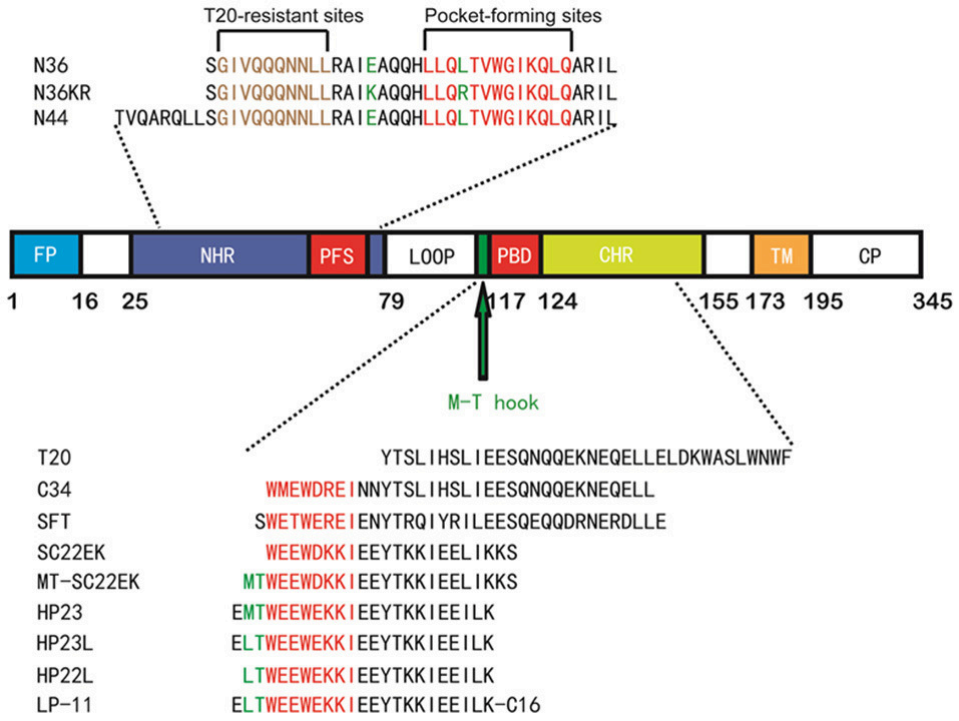
Schematic illustration of HIV-1 gp41 and its peptide fusion inhibitors. The functional domains of gp41 and the sequences of NHR or CHR-derived peptides are presented. The gp41 numbering of HIV-1 HXB2 is used. FP, fusion peptide; NHR, N-terminal heptad repeat; PFS, pocket-forming sites; PBD, pocket-binding domain; CHR, C-terminal heptad repeat; TM, transmembrane domain. The sequences of NHR- and CHR-derived peptides are listed. The sequence corresponding to the T20-resistant site is marked in brown; the sequences corresponding to the NHR pocket region and the pocket-binding domain (PBD) are marked in red. The position and sequence of the M-T or L-T hook structure as well as the E49K and L57R mutations are shown in green.

Peptides derived from the CHR or NHR regions of gp41 have potent anti-HIV activity by competitive binding to the counterpart (NHR or CHR) thus preventing the formation of viral 6-HB structure (Eggink et al., 2010; Steffen and Pohlmann, 2010; He, 2013). Enfuvirtide (T-20), a CHR-derived 36-mer peptide, is the first and only viral fusion inhibitor used in combination therapy of HIV-1 infection; however, its clinical use has been significantly limited owing to its high dosage and drug-resistance, thus calling for new HIV-1 fusion inhibitors with improved pharmaceutical properties (Baldwin et al., 2004; Greenberg and Cammack, 2004; Ashkenazi et al., 2011; Berkhout et al., 2012). In recent years, a number of new peptide fusion inhibitors, such as Sifuvirtide (He et al., 2008c), SC34EK and SC29EK (Otaka et al., 2002; Naito et al., 2009), and T2635 (Dwyer et al., 2007), have been generated by using the CHR peptide C34 as a design template (Figure 1), but they usually inherit long peptide sequences that also bind to the NHR region mediating T-20 resistance (Nameki et al., 2005; Eggink et al., 2008, 2011; Shimura et al., 2010; Liu et al., 2011).

The deep hydrophobic pocket on the C-terminal portion of the NHR helices has been considered an ideal drug target; however, the small molecules or short-peptides specifically targeting this site usually have weak binding affinity and thus, display low anti-HIV activity (Chan and Kim, 1998; Chan et al., 1998; Eckert et al., 1999; Jiang et al., 2004; Welch et al., 2007; Yu et al., 2014). Recently, we have demonstrated that the M-T hook structure, which is formed by two N-terminal residues (Met-115 and Thr-116) preceding the pocket-binding domain (PBD) of CHR peptides, can greatly improve the function of a short-peptide fusion inhibitor, providing a new strategy to develop anti-HIV drugs that block viral fusion step (He et al., 2008a,b; Chong et al., 2012a,b, 2013, 2014a,b, 2015a). We initially designed a 24-residue peptide termed MT-SC22EK by directly adding two hook residues to the N-terminus of the poorly active short-peptide SC22EK, which did show dramatically increased binding and antiviral activities (Chong et al., 2013). By referring the crystal structures of both SC22EK and MT-SC22EK, we further generated a highly potent short-peptide inhibitor termed HP23 (Chong et al., 2015b). To improve the pharmaceutical properties of a short-peptide-based inhibitor, the methionine residue in the M-T hook structure of HP23 was substituted by leucine that rendered less susceptibility to oxidation, resulting in a new inhibitor termed HP23L (Chong et al., 2016). Further, a panel of lipopeptide-based inhibitors were created by conjugating different lipids to the C-terminus of HP23L through a polyethylene glycol (PEG) linker, and of them a fatty acid-modified lipopeptide, termed LP-11, demonstrated the robust and long-lasting anti-HIV activity (Chong et al., 2016). Meanwhile, we selected a large panel of HIV-1 escape mutants that conferred high cross-resistance to the short-peptide inhibitors including SC22EK, MT-SC22EK, HP23, HP23L, and LP-11, and found that two mutations (E49K and L57R) at the inhibitor-binding site critically determined the resistance phenotypes either singly or in combination (Su et al., 2015a,b).

In the present study, we dedicated our efforts to elucidate the mechanism of action of our newly-designed short-peptide HIV-1 fusion inhibitors and the molecular basis underlying the drug-resistance. Thus, we determined the crystal structures of HP23L and LP-11 bound to a target-mimic NHR peptide with the wild-type sequence or resistant mutations. We also tried to resolve the structural properties of the isolated HP23L and LP-11 inhibitors. The resulted data have provided important information for understanding the structure and function of short-peptide HIV-1 fusion inhibitors that mainly target the gp41 pocket site and definitely will facilitate the development of novel antiviral drugs that block the viral fusion step.

## Materials and medthods

### Peptide synthesis and lipid conjugation

The peptides HP23L, N36, N36KR, and N44 were synthesized on rink amide 4-methylbenzhydrylamine (MBHA) resin by using a standard solid-phase 9-flurorenylmethoxycarbonyl (FMOC) method as described previously (Chong et al., 2016). All peptides were acetylated at the N-terminus and amidated at the C-terminus. For the lipopeptide LP-11, the template peptide HP23L contains lysine at its C-terminus with a 1-(4,4-dimethyl-2,6-dioxocyclohexylidene)ethyl (Dde) side-chain-protecting group, enabling the conjugation of a fatty acid (C16) that requires a deprotection step in a solution of 2% hydrazinehydrate-N,N-dimethylformamide (DMF) (Chong et al., 2016). Peptides were purified by reverse-phase high-performance liquid chromatography (HPLC) to more than 95% homogeneity and were characterized by mass spectrometry. Concentrations of the peptides were measured by UV absorbance and a theoretically calculated molar extinction coefficient based on the tryptophan and tyrosine residues.

### Assembly and crystallization of 6-HBs

The 6-HBs were assembled by dissolving equal amounts (1:1 molecular ratio) of the peptides (HP23L and N36; LP-11 and N44; or HP23L and N36KR) in denaturing buffer (100 mM NaH_2_PO_4_; 10 mM Tris-HCl, pH 8.0; and 8 M urea). To refold the peptides, the mixture was dialyzed against buffer containing 50 mM Tris-HCl (pH 7.5) and 100 mM NaCl at 4°C overnight. The dialyzed sample was concentrated by centrifugation and then subjected to the size-exclusion chromatography (Superdex 75 10/300 GL, GE Healthcare). Elutions corresponding to the molecular weight of a 6-HB were collected and concentrated prior to the crystallization trials. The complex (HP23L/N36; LP-11/N44; or HP23L/N36KR) was crystallized by mixing equal volumes (0.2 μl) of purified sample (~10 mg/ml) and the reservoir solution containing 0.1 M Tris-HCl (pH 8.5) and 10% (w/v) PEG4000) in a sitting drop vapor diffusion system at 18°C. The cryocooling for the crystals was achieved by soaking the crystal 5 s in the reservoir solution containing 30% (v/v) glycerol, followed by flash freezing to 100 K in liquid nitrogen. All data sets were collected on beamline BL17U at the Shanghai Synchrotron Research Facility (SSRF) and processed with HKL2000 (Otwinowski and Minor, 1997). All data collection and processing statistics were listed in Table 1.

**Table 1:** Data collection and refinement.

	**HP23L/N36 (PDB ID: 5YB3)**	**HP23L/N36KR (PDB ID: 5YB4)**	**LP-11/N44 (PDB ID: 5YB2)**
**DATA COLLECTION**			
Beamline	SSRF BL17U	SSRF BL17U	SSRF BL17U
Wavelength	0.9796 Å	0.9796 Å	0.9796 Å
Resolution range	36.17–2.04 (2.12–2.0)	35.15–2.5 (2.59–2.5)	27.72–3.8 (3.94–3.8)
Space group	*P* 4_3_2_1_2	*P* 4_1_2_1_2	*R* 3
Unit cell	51.15 51.15 168.74 90 90 90	51.29 51.29 142.89 90 90 90	110.88110.88125.38 90 90 120
Redundancy	18.5	12.4	5.7
Total reflections	2,78,824	90,820	40,763
Unique reflections	15003 (1442)	7140 (693)	5647 (556)
Completeness (%)	99.4(90.8)	100 (99.9)	100 (100)
R-merge (%)	12.7 (54.7)	10.3 (55.9)	10.8 (56.1)
I/σI	26.5 (7.1)	15.8 (5.0)	14.4 (3.5)
**REFINEMENT**			
Reflections used in refinement	14,998	7,128	5,646
R-work	0.187	0.188	0.282
R-free	0.231	0.266	0.306
Number of non-hydrogen atoms	1,605	1,503	3,495
Macromolecules	1,497	1,480	3,495
Protein residues	176	173	411
RMS bonds (Å)	0.007	0.008	0.021
RMS angles (°)	0.77	0.90	1.75
Ramachandran favored (%)	100	98	96
Ramachandran allowed (%)	0	1.2	3.1
Ramachandran outliers (%)	0	0.62	0.78
Rotamer outliers (%)	1.9	5.1	6.9
Clashscore	3.94	8.56	24.67
Average B-factor (Å^2^)	27.25	54.40	134.31
Macromolecules	26.47	54.47	134.31
Solvent	38.06	50.24	

*R_work_ and R_free_ are defined by R = Σ_hkl_||F_obs_| – |F_calc_||/Σ_hkl_|F_obs_|, where h, k, and l are the indices of the reflections (used in refinement for R_work_; 5%, not used in refinement for R_free_) and F_obs_ and F_calc_ are the structure factors, deduced from intensities and calculated from the model, respectively*.

### Structural determination and refinement

The crystal structures of HP23L/N36, LP-11/N44, and HP23L/N36KR were solved by molecular replacement with the crystallographic software PHASER (McCoy et al., 2007). The searching model was the MT-SC22EK/T21 structure (PDB ID: 3VU6). The iterative refinement with the program PHENIX (Adams et al., 2002) and model building with the program COOT (Emsley and Cowtan, 2004) were performed to complete the structure refinement. Structure validation was performed with the program PROCHECK (Laskowski et al., 1993), and all structural figures were generated with PyMOL (http://www.pymol.org).

### Circular dichroism (CD) spectroscopy

CD spectroscopy was conducted according to our protocol described previously (Chong et al., 2016). Briefly, a CHR peptide (HP23L or HP22L) was incubated with an equal molar concentration (10 μM) of the NHR peptide N36 at 37°C for 30 min in PBS (pH 7.2). CD spectra were acquired on a Jasco spectropolarimeter (model J-815) using a 1 nm bandwidth with a 1 nm step resolution from 195 to 270 nm at room temperature. Spectra were corrected by subtraction of a solvent blank. The α-helical content was calculated from the CD signal by dividing the mean residue ellipticity [θ] at 222 nm by the value expected for 100% helix formation (−33,000 degree.cm^2^.dmol^−1^). Thermal denaturation was performed by monitoring the ellipticity change at 222 nm from 20 to 98°C at a rate of 2°C/min, and *T*_*m*_ (melting temperature) was defined as the midpoint of the thermal unfolding transition.

### Cell–cell fusion assay

Activity of inhibitors on HIV-1 Env-mediated cell-cell fusion was measured using a dual split protein (DSP)-base assay as described previously (Ishikawa et al., 2012; Chong et al., 2017). Briefly, 293T cells (effector cells) were plated in 96-well plate (1.5 × 10^4^/well) and incubated at 37°C. On the following day, 293T cells were transfected with a mixture of an HIV-1NL4-3 Env-expressing plasmid and a DSP_1−7_ plasmid. Twenty-four hours posttransfection, 293FT cells stably expressing CXCR4/CCR5 and DSP_8−11_ (target cells) were resuspended and added EnduRen live cell substrate, followed by incubation of 30 min at 37°C. Then, the target cells (3 × 10^4^/well) were co-cultured with effector cells at 37°C in the presence or absence of a tested inhibitor at graded concentrations. The mixed cells were then spun down to maximize cell-cell contact and incubated for 1 h at 37°C. Luciferase activity was measured using luciferase assay regents and a luminescence counter (Promega, Madison, WI, USA).

### Single-cycle infection assay

HIV-1 entry and its inhibition were measured by single-cycle infection assay as described previously (Chong et al., 2017). Briefly, HIV-1_NL4−3_ pseudoviruses were generated via cotransfection of 293T cells with an Env-expressing plasmid and the backbone plasmid pSG3Δenv containing an Env-defective, luciferase-expressing HIV-1 genome. Culture supernatants were harvested 48 h after transfection, and 50% tissue culture infectious doses (TCID_50_) were determined in TZM-bl cells. Peptides were prepared in 3-fold dilutions and mixed with 100 TCID_50_ of pseudoviruses, and then incubated 1 h at room temperature. The mixture was added to TZM-bl cells (10^4^/well) and incubated for 48 h at 37°C. The luciferase activity was measured using luciferase assay reagents and a luminescence counter (Promega).

## Results

### Crystallization and structure determination of HP23L/N36 complex

To dissect the molecular mechanism of action of the potent inhibitor HP23L, we assembled and crystallized the complex of HP23L and N36, an NHR-derived target mimic peptide. Two peptides were equally dissolved in denaturing buffer, and the mixture was dialyzed to allow refolding of the peptides. Then, the HP23L/N36 complex was purified by size-exclusion chromatography and was crystallized using commercial kits. The crystal of the HP23L/N36 complex belonged to the space group of *P*4_1_2_1_2, contained three pairs of HP23L/N36 peptides (one complete 6-HB) per asymmetric unit, and diffracted x-ray to a resolution limit of 2.0 Å (Protein Data Bank [PDB] accession number: 5YB3). We could build all the residues of the HP23L/N36 peptides in the electronic density map except the extremely N-terminal residue Ser-35 on one chain of the N36 trimer. The refined model has good refinement statistics and stereochemistry quality (Table 1).

### General features of HP23L/N36-based 6-HB structure

**Figure 2:**
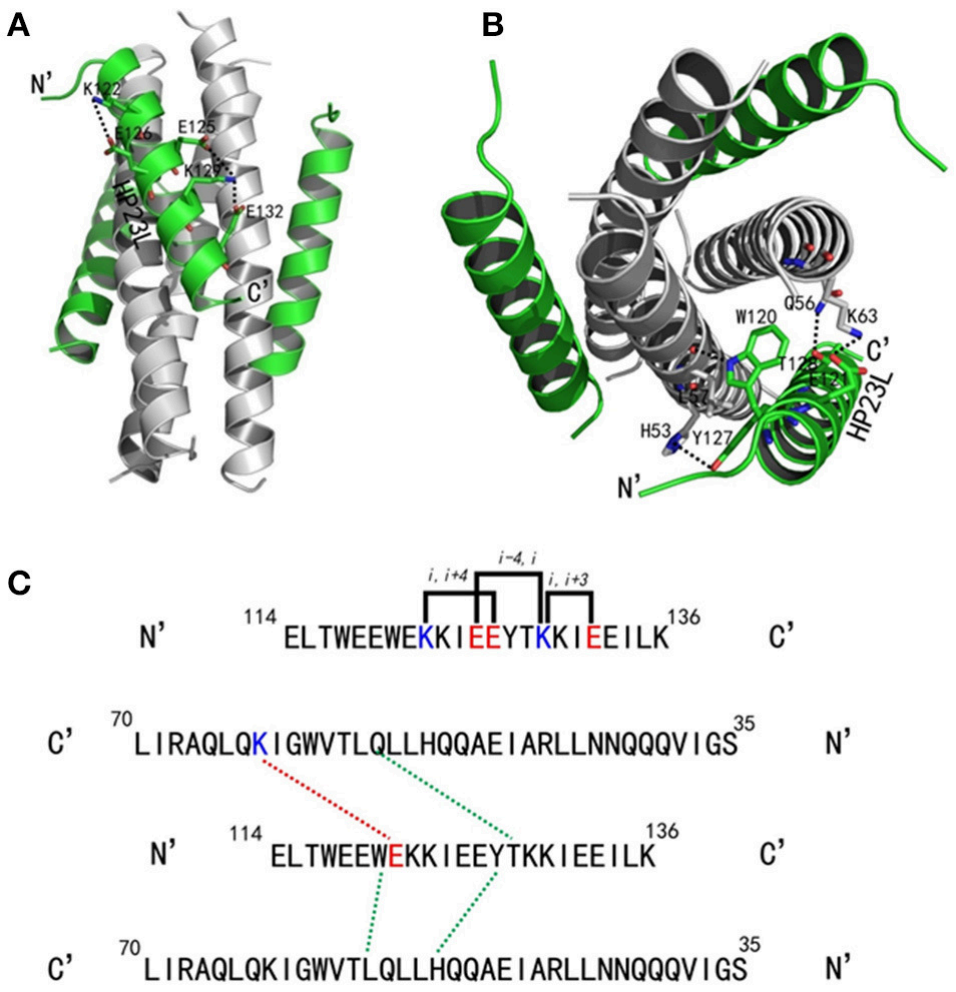
Salt-bridges and hydrogen bonds in 6-HB structure of HP23L/N36. **(A)** A ribbon model of the 6-HB structure formed by HP23L/N36. The N36 trimer is colored in gray and the HP23L peptides are colored in green. The charged residues involving ion pair formation on the HP23L helix are shown as stick models with labels. The salt-bridges formed between charged residues are indicated in dashed lines. **(B)** A ribbon model of the 6-HB structure formed by HP23L/N36. The N36 trimer is colored in gray and the HP23L peptides are colored in green. The residues involving the formation of ion pair and hydrogen bonds between HP23L and N36 trimer are shown as stick models with labels. The salt-bridge and hydrogen bonds formed between residues are indicated with dashed lines. **(C)** Sequence illustration of salt-bridges and hydrogen bonds in the 6-HB of HP23L/N36. The upper indicates the intrahelical salt-bridges of HP23L in solid black lines, while the positively charged lysine and the negatively charged glutamic are respectively marked in blue and red. The lower indicates a single HP23L peptide interacting with two N36 helices, in which the dashed red line indicates a salt-bridge between Lys-63 on N36 and Glu-121 on HP23L, and the dashed green lines indicate the interhelical hydrogen bonds.

As anticipated, HP23L and N36 formed a typical 6-HB structure similar to many other gp41 core structures (Figure 2A). In highlight, three N36 helices formed an interior, trimeric coiled coil with three conserved, hydrophobic grooves, and three HP23L inhibitor helices packed into each of the grooves in an antiparallel manner. Typically, a deep pocket was presented at the basis of the NHR grooves, which was formed by about 11 residues and nearly 16 Å long, 7 Å wide, and 5–6 Å deep. To gain a stable helical conformation of HP23L, the charged residues glutamic acid and lysine (EK motif) were introduced to promote the formation of intra-helical salt-bridges. The crystal structure showed that the ion pairing did not form as the original design, but revealed three salt-bridges between the oppositely charged residues that stabilized the α-helical conformation of the inhibitors (Figure 2A). Specifically, the positively charged side chain of Lys-122 and the negatively charged side chain of Glu-126 attracted with each other to form a salt-bridge at *i* and *i* + 4 positions, thus stabilizing the upstream helix of HP23L. More interestingly, the positively charged Lys-129 positioned its long side chain to pair with the upstream Glu-125 and the downstream Glu-132 simultaneously to form two salt-bridges at *i* and *i* + 4 or *i* + 3 positions, respectively. From a perspective along the helix axis, it could be easily observed that Lys-122 and Glu-126 at one side of the α-helix of HP23L stabilized the N-terminus of the inhibitors, and the salt-bridges between Lys-129 and Glu-125, Lys-129 and Glu-132 at the other side of the α-helix of HP23L stabilized the C-terminus of the inhibitors. Therefore, the salt-bridges of HP23L evenly distributed at both sides of its α-helix balanced the interior force of the inhibitors thus making the conformation stable.

The inter-helical hydrogen bonds and salt-bridges between HP23L and N36 also critically determined the binding of the inhibitors (Figure 2B). Specifically, the Nε1 atom of Trp-120 in the PBD of HP23L donated a hydrogen bond to the O atom of Leu-57 in the pocket of the N36 trimer in addition to its hydrophobic interactions with multiple pocket-forming residues; the OH group of Tyr-127 of HP23L accepted a hydrogen bond from the Nδ1 group of His-53 of the N36 trimer and the Oγ1 atom of Thr-128 of HP23L accepted a hydrogen bond from the Nε2 atom of Gln-56 on N36 trimer; the Oε1 atom of the negatively charged side chain of Glu-121 on HP23L attracted the Nζ atom of the positively charged side chain of Lys-63 on N36 trimer forming an inter-helical salt-bridge further strengthening the stability of 6-HB. Therefore, the hydrogen bonds and salt-bridges formed with the N36 trimer at both left and right sides of the HP23L inhibitors simultaneously should have extremely increased the stability of the 6-HB structure (Figure 2C).

**Figure 3:**
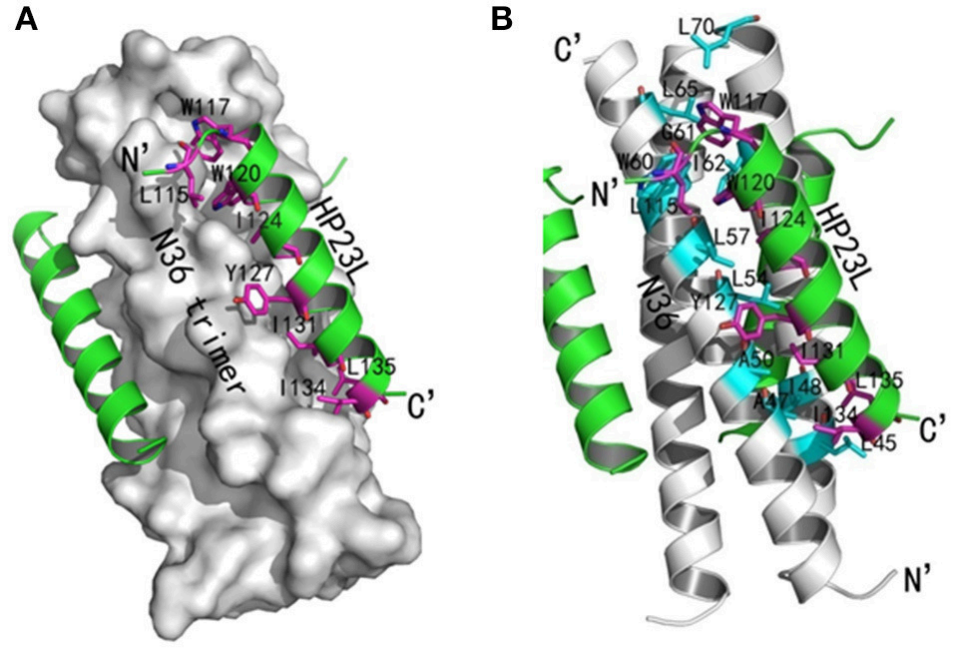
Hydrophobic interactions in 6-HB structure of HP23L/N36. **(A)** The crystal structure of HP23L/N36 is presented in ribbon and surface models vertically by PyMOL. The N36 trimer is shown as surface model and colored in gray. The HP23L inhibitors are shown as ribbon models and colored in green. Residues related to hydrophobic interactions with N36 trimer on HP23L are shown as stick models in pink. The N- and C-termini of HP23L are labeled. **(B)** The crystal structure of HP23L/N36 is presented in ribbon models vertically by PyMOL. The N36 trimer is shown in gray. Residues related to hydrophobic interactions on N36 trimer are shown as stick models and colored in cyan. HP23L inhibitors are colored in green. Residues related to hydrophobic interactions with N36 trimer on HP23L are shown as stick models in pink.

Similar to other 6-HB structures of the gp41 core, abundant hydrophobic interactions played vital roles to stabilize the 6-HB structure. First, three hydrophobic residues from the PBD of HP23L (Trp-117, Trp-120, and Ile-124) inserted into the deep pocket of N36 trimer and made extensive hydrophobic contacts with the pocket-forming residues (Figure 3A). Specifically, Trp-117 interacted with Gly-61, Leu-65, Leu-70, and Ile-62; Trp-120 interacted with Trp-60, Leu-57 and Ile-62; and Ile-124 interacted with Leu-57 and Leu-54. Second, it is worth noting that Tyr-127 in the middle region of HP23L not only had hydrophobic interaction with Leu-54 but also interacted with Leu-57 at the rim of the hydrophobic pocket of the N36 trimer. Third, the downstream hydrophobic residues of HP23L were also critically involved in the hydrophobic network. For example, Ile-131 interacted simultaneously with Ala-50 and Leu-54, Ile-134 interacted with Ala-47, and Leu-135 contacted simultaneously with Leu-45 and Ile-48, which were located immediately at the pocket upstream of the N36 trimer (Figure 3B).

### The N-terminal hook-like conformation greatly stabilizes the 6-HB structure

**Figure 4:**
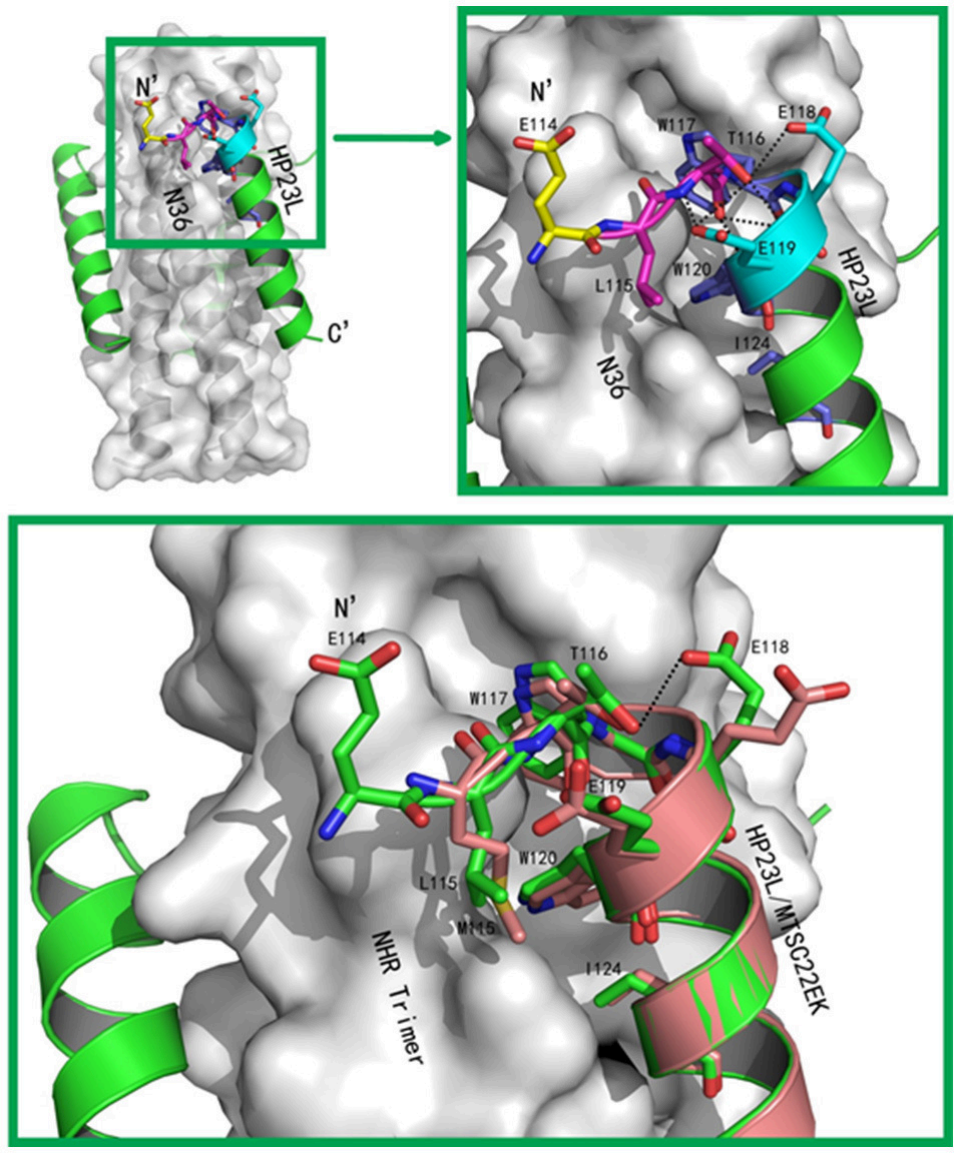
Visualization of the L-T hook conformation of HP23L**. (A)** Crystal structure of HP23L/N36 is presented in ribbon and surface models. N36 trimer is in gray. Residues in the L-T hook region and pocket-binding domain are respectively colored in pink and blue in stick models. The extreme N-terminal glutamic is shown in yellow with labels, while other glutamic residues are shown as stick models in cyan with labels. Hydrogen bonds existed in L-T hook region are indicated in dashed lines. **(B)** The region of hydrophobic bond network of the L-T hook on HP23L is zoomed. The Hydrogen bonds in L-T hook region are indicated in dashed lines with respective distances. **(C)** The L-T hook of HP23L and the M-T hook of MT-SC22EK are superimposed for comparison. N36 and T21 trimers are shown as surface models in gray. HP23L and MT-SC22EK are shown as ribbon models and colored in green and salmon, respectively. The L-T hook region and pocket-binding domain are shown as stick models with labels. Hydrogen bonds existed in N-termini of HP23L are indicated in dashed black lines.

The conformations of all N-terminal residues in HP23L were defined in the electron density map, indicating that the N terminus of the peptide was stable. Importantly, Leu-115 and Thr-116 at the upstream of the PBD adopted a hook-like (designated as L-T hook) structure similar to the M-T hook structure observed in other crystal structures (Figures 4A,B), such as MT-C34, MT-SFT and MT-SC22EK (Chong et al., 2012a, 2013, 2014b). Briefly, Thr-116 terminated the α-helical conformation of HP23L by spinning its dihedral angle Ψ by nearly 180°, resulting in the N-terminus of HP23L kept away from the central coiled-coil trimer. The side chain of upstream Leu-115 was settled at the top of the left side of the hydrophobic pocket on the N36 trimer, so that the hydrophobic side chain of Leu-115 accommodated the hydrophobic groove between N36 and HP23L helices, thus covering the hydrophobic pocket below. Thus, Leu-115 had huge hydrophobic interactions with multiple pocket-forming residues such as Trp-60 and Leu-57, strengthening the stability of L-T hook structure and the binding of inhibitors.

Besides the hydrophobic contacts, hydrogen bonds in the L-T hook region also increased the binding stability of the hook and pocket region (Figure 4C). Interestingly, the carbonyl group of Thr-116 formed hydrogen bonds with the backbone NH groups of Glu-119 (distance = 3.10 Å, angle = 110.87°) and Trp-120 (distance = 2.89 Å, angle = 162.79°), respectively, while the hydroxyl group of Thr-116 also formed a hydrogen bond with the backbone of Glu-119 (distance = 3.05 Å, angle = 162.55°); the Oγ1 atom of the side chain of Thr-116 formed a hydrogen bond with the Oε1 atom of the side chain of Glu-118 (distance = 3.66 Å, angle = 122.57°) and with Oε1 atom of the side chain of Glu-119 (distance = 3.47 Å, angle = 103.05°), respectively. Also importantly, the Oε1 atom of the side chain of Glu-119 accepted a strong hydrogen bond from the backbone NH group of Thr-116 (distance = 2.9 Å, angle = 167.99°), which further stabilized the conformation of L-T hook structure and strengthened hydrophobic binding of the upstream Leu-115 with the hydrophobic pocket on the NHR trimer.

### Addition of an extra glutamic residue stabilizes the L-T hook structure and increases the inhibitory activity

In the original design of HP23L, an extra glutamic acid (Glu-114) was added to the extreme N-terminus of the peptide to enhance the stability of the inhibitors. Surprisingly, except for stability of the inhibitor itself, the structure verified that the backbone of glutamic (Glu-114) did not follow the direction of the α-helix but twisted its dihedral angle Ψ by nearly 180°, thus holding the NHR trimer inside and its carboxylic side chain climbed onto the wall of the NHR trimer increasing the contacts (Figures 4A,B). Therefore, the Glu-114 could enhance the binding potency of the inhibitor with the NHR trimer.

**Figure 5:**
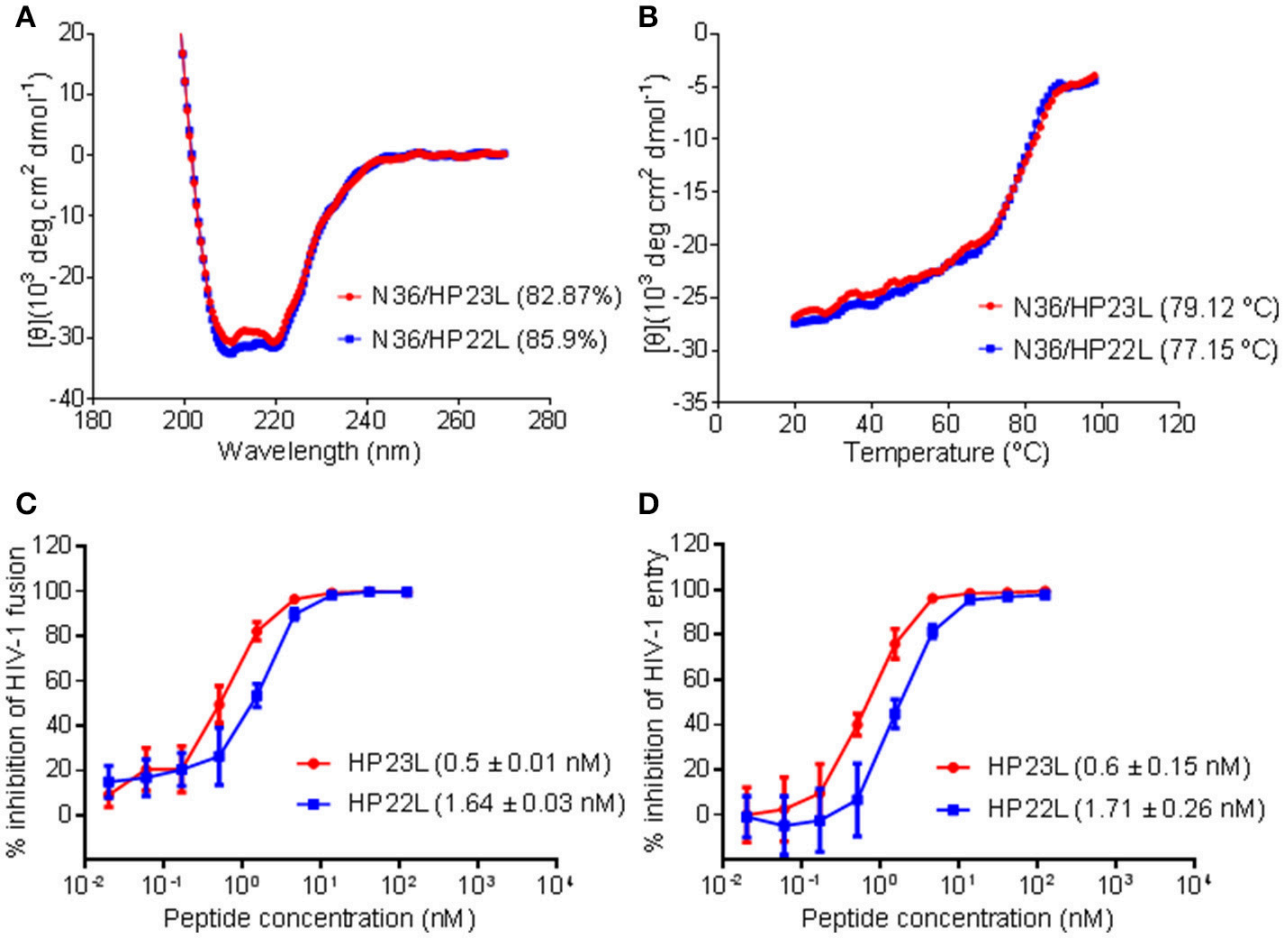
Biophysical and functional characterization of the extra N-terminal glutamic residue of HP23L. The α-helicity **(A)** and thermostability **(B)** of HP23L- and HP22L-based 6-HBs were compared by CD spectroscopy. The final concentration of each peptide in PBS was 10 μM. The % helical content and *T*_m_ values are shown in parentheses. The inhibitory activities of HP23L and HP22L on HIV-1 Env-mediated cell-cell fusion **(C)** and pseudovirus entry **(D)** were measured by DSP assay and single cycle infection assay, respectively. The experiments were repeated 3 times, and mean IC_50_ values are shown in parentheses.

To verify the results received from the structural data, we synthesized the peptide HP22L by deleting the N-terminal Glu-114 from HP23L and then compared their binding and inhibitory activities. First, the α-helicity and thermal stability of HP22L and HP23L paired with N36 were determined by CD spectroscopy. As shown in Figures 5A,B, the HP22L/N36-based 6-HB exhibited a *T*_m_ value of 77.15°C, while the HP23L/N36-based 6-HB showed a *T*_m_ value of 79.12°C, indicating that the truncation of Glu-114 could reduce the binding stability of the inhibitor. Next, we compared the inhibitory activity of HP22L and HP23L. In cell-cell fusion assay, HP22L and HP23L inhibited HIV-1_NL4−3_ Env-mediated cell fusion with IC_50_ values of 1.64 and 0.5 nM, respectively, indicating a 3.28-fold decrease for HP22L relative to HP23L (Figure 5C). In single-cycle infection assay, HP22L and HP23L inhibited HIV-1_NL4−3_ pseudovirus with IC_50_ values of 1.71 and 0.6 nM, respectively, indicating a 2.85-fold decrease for HP22L (Figure 5D). Therefore, the structural and functional data confirmed the importance of adding a single extra glutamic acid to the N-terminus of the L-T hook structure.

### Crystal structure of the lipopeptide inhibitor LP-11 bound to N44

As a lipopeptide, LP-11 exhibited sharply increased binding stability and anti-HIV activity relative to its template HP23L (Chong et al., 2016). We were interested to know the molecular basis underlying the improved functionalities of LP-11. Thus, we determined its crystal structure in complex with an N-terminally extended NHR peptide, N44. Similarly, the LP-11/N44 complex was assembled, purified and crystallized. The crystal of the LP-11/N44 belonged to the space group of *R*3 contained seven pairs of LP-11/N44 peptides (two complete 6-HBs and a one third 6-HB) per asymmetric unit, and diffracted x-ray to a resolution limit of 3.8 Å (PDB accession number: 5YB2). Although the resolution of this structure was relatively low, we could build the most residues of LP-11/N44 in the electron density map except for eight overhung residues at the N-terminus of N44, which were not targeted by LP-11 thus their missing did little for inhibitor binding. As predicted, the C-terminal fatty acid group of LP-11 could not be observed due to the poor electron density map. As shown in Table 1, the refined model has good refinement statistics and stereochemistry quality.

**Figure 6:**
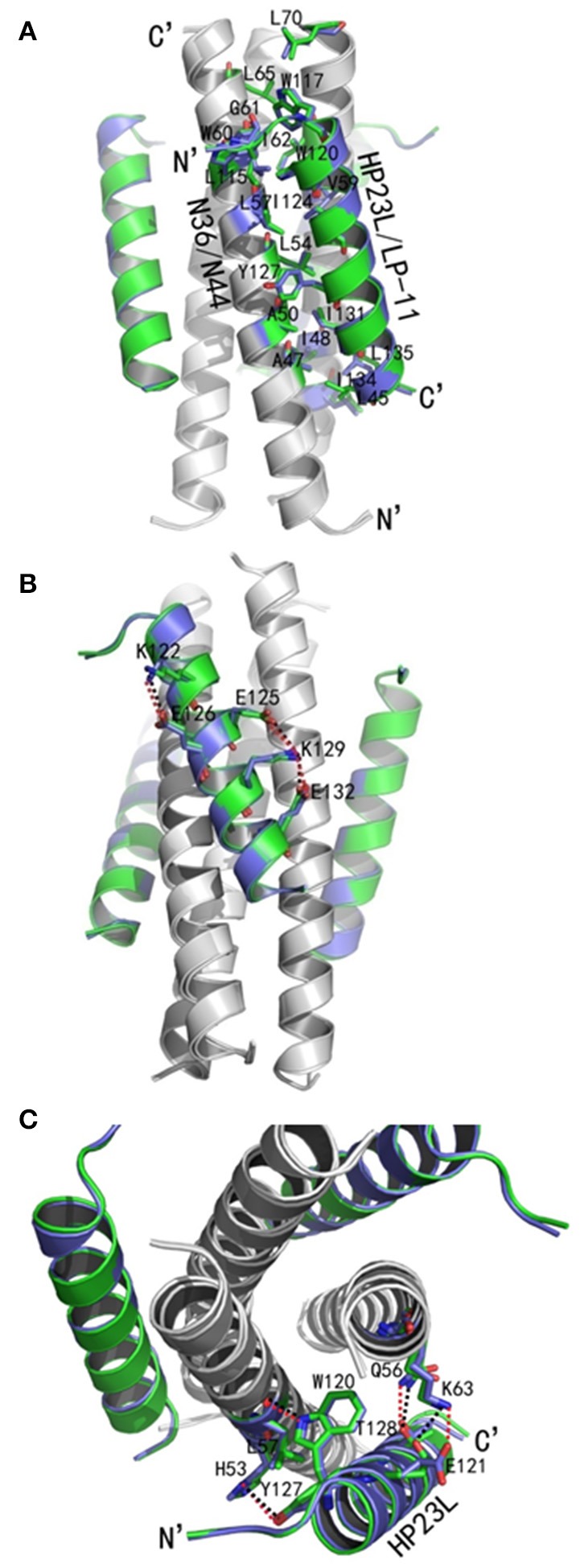
Crystal structure of 6-HB formed by LP-11 and N44. **(A)** The structures of HP23L/N36 and LP-11/N44 are superimposed in ribbon models and compared for hydrophobic interactions. N44 and N36 trimers are shown in gray, HP23L and LP-11 are respectively shown in green and blue. Residues related to hydrophobic interactions of HP23L/N36 are shown as stick models and marked in green with labels, while the residues related to hydrophobic interactions of LP-11/N44 are shown as stick models and marked in blue with labels. **(B)** Superimposing comparison of the intrahelical salt-brides of HP23L and LP-11. NHR trimer is shown in gray; HP23L and LP-11 are respectively shown in green and blue. Residues involving in the formation of salt-bridges are shown as stick models with labels. Salt-bridges of HP23L and LP-11 are respectively indicated with dashed black and red lines. **(C)** Superimposing comparison of the interhelical salt-brides and hydrogen bonds of LP-11/N44 and HP23L/N36. NHR trimer is shown in gray. HP23L and LP-11 are respectively shown in green and blue. Residues involving in the salt-bridges and hydrogen bonds are shown as stick models with labels. Salt-bridges and hydrogen bonds between HP23L and N36 are indicated with dashed black lines. Salt-bridges and hydrogen bonds between LP-11 and LP-11 are indicated with dashed red lines.

The crystal structure of LP-11/N44 verified a typical 6-HB conformation, in which trimeric central N44-based coiled coils were bound by three inhibitor peptides in an antiparallel orientation, and they made a plenty of hydrophobic interactions similar to the 6-HB structure of HP23L/N36 (Figure 6A). However, two structures revealed the major differences in hydrophobic contacts. First, Trp-120 of LP-11 did not have any contacts with Ile-62 but had hydrophobic contacts with Val-59 on N44 trimer in addition to contacting with Trp-60 and Leu-57. Second, Ile-134 of LP-11 failed to contact with Ala-47 while its Leu-135 failed to contact with Ile-48 on N44 trimer, which were found possessing hydrophobic interactions in the HP23L/N36-based 6-HB structure.

**Figure 7:**
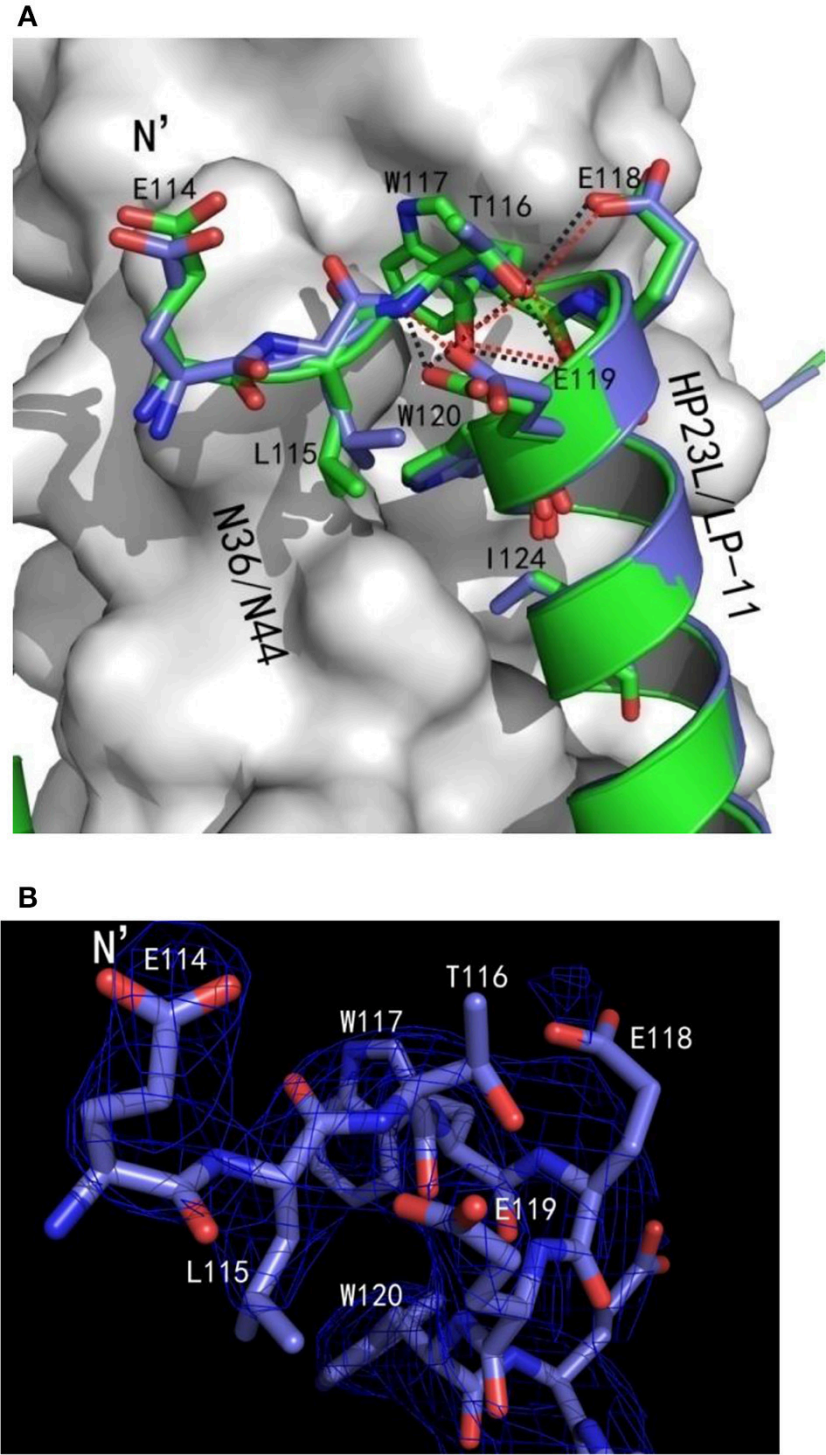
Comparison of the hook regions of HP23L and LP-11 by superimposing. **(A)** N44 and N36 trimers are shown as surface models in gray. HP23L and LP-11 are respectively shown in green and blue. Residues involving in the hook and pocket-binding domain are shown as stick models with labels. Hydrogen bonds existed in N termini of HP23L and LP-11 are indicated in dashed lines and shown respectively in black and red. **(B)** The hook region of LP-11 is shown with the final model with the superimposed 2Fo-Fc electron density map (1.5 s contour, blue mesh). Stick model is colored with the same scheme used in **(A)**.

Surprisingly, there were exactly the same salt-bridges and hydrogen bonds in the two 6-HB structures. For example, three pairs of intrahelical salt-bridges (Lys-122/Glu-126, Lys-129/Glu-125, Lys-129/Glu-132) were also identified on LP-11 itself (Figure 6B); the Oε2 atom of the negative charged side chain of Glu-121 of LP-11 was attracted by the Nζ atom of the positive charged side chain of Lys-63 on N44 trimer forming an interhelical salt-bridge; the NH group of Trp-120 of LP-11 donated a hydrogen bond to the OH group of the Leu-57 of the N44 trimer; the OH group of Tyr-127 of LP-11 accepted a hydrogen bond from the N group of His-53 of the N44 trimer, and the Oγ1 atom of Thr-128 of LP-11 accepted a hydrogen bond from the Nε2 atom of Gln-56 of the N44 trimer to increase the binding of N44 and LP-11 at the other side of the inhibitors (Figure 6C). Importantly, the superimposing comparison demonstrated that the L-T hook structure in the 6-HB of LP-11/N44 was exactly identical to that of HP23L/N36, including its intra-helical hydrogen bond network and inter-helical hydrophobic interactions (Figure 7).

### Structural insights of HIV-1 resistance to fusion inhibitors targeting the gp41 pocket

We previously discovered that the mutations E49K and L57R in inhibitor-binding site of NHR conferred high resistance to the short-peptide inhibitors that contained the M-T or L-T hook structure and specifically targeted the hydrophobic pocket (Su et al., 2015a,b). Here, we sought to dissect the molecular basis involved in the resistance phenotypes by crystallographic studies. To this end, the peptide N36 with the E49K and L57R substitutions (designated N36KR) was synthesized and used to assemble the complex with HP23L for crystallization. The crystal of HP23L/N36KR belonged to the space group of *P*4_1_2_1_2, contained three pairs of HP23L/N36KR peptides (one complete 6-HB) per asymmetric unit, and diffracted x-ray to a resolution limit of 2.5 Å (PDB accession number: 5YB4). Similarly, we could build all residues of the HP23L/N36KR peptides in the electron density map except several residues unrelated to resistance analysis. The sequence of N36KR was built from Ser-35 to Leu-70 on two chains and from Gly-36 to Leu-70 on another chain of the N36KR trimer; the sequence of HP23L was built from Thr-116 to Lys-136 on one chain and from Leu-115 to Lys-136 on the second chain and from Glu-114 to Lys-136 on the third chain of the N36 trimer. The refined model has good refinement statistics and stereochemistry quality (Table 1).

**Figure 8:**
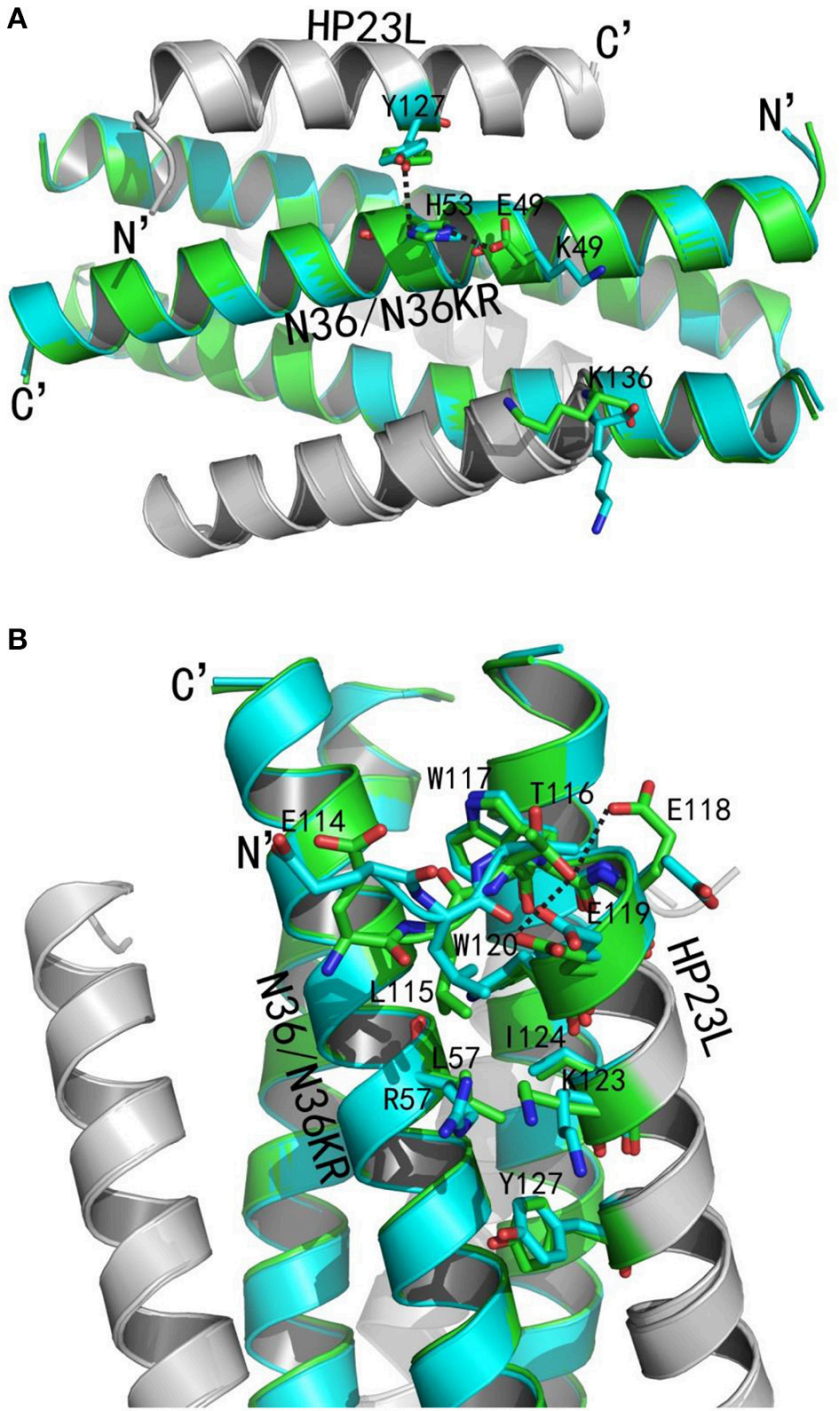
Revealing the mechanism of HIV-1 resistance by crystal structure. The structures of HP23L bound to N36 or N36 with E49K/L57R mutations (N36KR) are superimposed for comparison. N36 and N36KR are respectively shown in green and cyan. HP23L inhibitors are shown in gray. **(A)** Analysis of E49K-mediated resistance. The residues related to the resistant site are shown as stick models with labels. The salt-bridge and hydrogen bonds are indicated in dashed black lines. **(B)** Analysis of L57R-mediated resistance. The N-trimer, inhibitors, and the residues related to the resistant site are shown and labeled as **(A)**. A hydrogen bond in the hook region is indicated in dashed black lines.

We analyzed the effects of the E49K and L57R substitutions by comparing the data of crystal structures of HP23L/N36 and HP23L/N36KR (Figures 8A,B). In the 6-HB structure of HP23L/N36, Oε1 atom of the side chain of the negatively charged Glu-49 attracted Nε2 atom of the side chain of the positively charged His-53 forming a strong salt-bridge with a distance of 2.68 Å contributing the α-helical conformation. Further, Nζ1 atom of the side chain of His-53 on N36 trimer formed a hydrogen bond (distance = 3.55 Å) with the OH group of the side chain of Tyr-127 on HP23L inhibitors stabilizing the binding of inhibitors either. Therefore, the three residues, Glu-49 and His-53 on N36 trimer and Tyr-127 on inhibitors, connected sequentially by salt-bridges and hydrogen bonds increasing both the intrahelical and interhelical stability. However, the Lys-49 in the structure of HP23L/N36KR destroyed the salt bridge with His-53 thus damaged indirectly the hydrogen bond between His-53 on N36KR and Tyr-127 on inhibitor contributing the resistance. Further, the positively charged Lys-49 repelled away the side chain of the same charged Lys-136 located at the extreme C-terminus of HP23L, which markedly impaired the binding of the inhibitors thus conferring the resistance. So the E49K mutation, together with its upstream His-53, downstream Arg-46 and Lys-136 on HP23L, greatly increased the positively charged repulsion near the mutation site, eventually resulting in instability of the 6-HB structure.

Clearly, the residue Leu-57 was located on the left wall of the deep pocket thus having huge hydrophobic interactions simultaneously with the residues from the L-T hook and the PBD of the HP23L inhibitors (Figure 8A). In other words, the Leu-57 was targeted by the L-T hook structure of HP23L, in which the side chain of Leu-57 physically interacted with the hydrophobic side chain of Leu-115 that fortified the binding of the pocket-binding residues of HP23L with the hydrophobic pockets. However, the substitution of L57R dramatically disrupted the hydrophobic interactions between Leu-115 and Leu-57. Moreover, the positively charged residue Arg-57 on N36KR attracted the negatively charged residue Glu-118 on inhibitors, which resulted in the side chain of Glu-118 twisted its dihedral angle Ψ by nearly 180°, thus failing to form the hydrogen bond with the Thr-116. Similarly, the Glu-119 also turned its side chain an angle toward Arg-57, thus it failed to form the two hydrogen bonds with the Thr-116. The disruption of three major hydrogen bonds in the hook region extremely decreased the NHR-binding stability of the inhibitors thus contributing the resistance. Also importantly, the Leu-57 had abundant hydrophobic interactions with the hydrophobic residues in the PBD of HP23L, such as Trp-120 and Ile-124, but the side chain of Arg-57 in the crystal structure of HP23L/N36KR was oriented away from the direction of the PBD and eliminated all of the hydrophobic interactions with the Trp-120 and Ile-124 of the inhibitors.

In addition to the L-T hook and PBD regions in the N-terminus of HP23L, the L57R substitution also lost its hydrophobic interaction with Tyr-127 in the middle of the inhibitor. Most importantly, the positively charged Arg-57 pushed the positively charged Lys-123 on inhibitors away increasing the repulsion between the NHR trimer and the inhibitors (Figure 8B). In summary, the L57R substitution eliminated the hydrophobic interactions between the resistant site and the N-terminal hydrophobic residues of inhibitors and disturbed the original conformation of Glu-118 and Glu-119, and eventually, the change of the conformation of Glu-118 and Glu-119 destroyed the hydrogen bonds in the L-T hook region thus loosing the acting force of the hook region of inhibitors. All of these weakened the binding stability of HP23L inhibitors thus determining the resistance.

## Discussion

In the present study, we have dedicated our efforts to determine the crystal structures of our newly-designed short-peptide HIV-1 fusion inhibitors that mainly target the deep pocket site of gp41, which provide important information for understanding their structure-activity relationship (SAR). First, we determined the crystal structures of HP23L and its lipid derivative LP-11 in complexes with the NHR-derived target peptides N36 or N44, which revealed their critical binding residues and motifs relative to the potent anti-HIV activity. Then, we determined the crystal structure of HP23L bound to N36 carrying two mutations (E49K and L57R) responsible for the drug-resistance, which further provided insights into the mechanisms of action of short-peptide viral fusion inhibitors.

Being the first member of a new class of anti-HIV drugs - HIV entry inhibitors, T-20 is an extremely useful addition to the arsenal of AIDS drugs and has been successful in treating HIV-1 infection failed to respond to current antiretroviral therapeutics targeting viral enzymes, including reverse transcriptase inhibitors (RTIs) and protease inhibitors (PIs) (Kilby et al., 1998; Lalezari et al., 2003; Matthews et al., 2004). However, T-20 has a relatively low genetic barrier to inducing drug-resistance rather easily both *in vivo* and *vitro*, and the emergence and the spread of diverse T-20-resistant HIV-1 mutants have significantly affected its effectiveness (Baldwin et al., 2004; Greenberg and Cammack, 2004; Sista et al., 2004; Xu et al., 2005; Chinnadurai et al., 2007). Mutations related to T-20 resistance are primarily mapped to the amino acids 36–45 of the inhibitor-binding site in the NHR of gp41, with the ^36^GIV_38_ motif being a hotspot (Rimsky et al., 1998; Baldwin et al., 2004; Heil et al., 2004; Sista et al., 2004; Chinnadurai et al., 2007; Eggink et al., 2009; Lobritz et al., 2010). Moreover, T-20-resistant HIV-1 mutants usually display cross-resistance to C34-based derivatives, because which also contain the binding sites at their C-terminals to the 36–45 region of NHR (Steffen and Pohlmann, 2010; Berkhout and Sanders, 2011; Berkhout et al., 2012; He, 2013). By adding the M-T hook residues (Met-115 and Thr-116) to the N-terminus of the short-peptides that specifically target the deep hydrophobic pocket rather than the T-20 resistant site, we have successfully designed fusion inhibitors with dramatically increased binding stability and inhibitory activity, such as MT-SC22EK (Chong et al., 2013), HP23 (Chong et al., 2015b), HP23L and LP-11 (Chong et al., 2016), 2P23 (Xiong et al., 2017), and LP-19 (Chong et al., 2017). Here, the crystal structures of HP23L and LP-11 bound to the target sequence have finely demonstrated the intra-helical and inter-helical interactions underlying the molecular basis of such inhibitors. Especially, the structural data indicate that the N-terminal methionine residue in the M-T hook structure of inhibitors can be replaced by the oxidation-less prone residue leucine, adopting a L-T hook conformation similar to the M-T hook structure presented in the inhibitors MT-SC22EK (Chong et al., 2013), CP32 (Chong et al., 2012b), MT-C34 (Chong et al., 2012a), and MT-SFT (Chong et al., 2014b), thus explaining why HP23L can retain its highly potent anti-HIV activity as does its template HP23. In comparison, the side chain of leucine in the L-T hook of HP23L is slightly shorter than the side chain of methionine in the M-T hook of MT-SC22EK (Figure 4C), but they show similar hydrophobic interactions with the deep pocket site on the NHR helices, illustrating that the hook-like structure can be readily retained in the N-terminal of inhibitors as long as the position 115 is placed a hydrophobic amino acid with a relatively long side chain. To this point, we are interested to know whether other residues can act similarly or even more promisingly when placing in the hook structure of inhibitors. Our structures also verify the importance of the extreme N-terminal glutamic acid of inhibitors in stabilizing the hook conformation and its binding stability with the pocket site. First, as an acidic amino acid added to the N-terminus of the peptides the Glu-114 stabilizes the α-helical conformation of HP23L and LP-11; second, the backbone of Glu-114 did not follow the direction of the α-helix but closed to the direction of N36 trimer and its side chain climbed onto the wall of the NHR trimer, being almost parallel with the α-helical backbone of inhibitors, like a hand to hold the N-trimer inside, thereby increasing the binding area to NHR-trimer. The calculated interface area between one HP23L and trimeric N36 was 1327.9–1401.4 Å^2^, where Glu-114 contributed 180.96–219.75 Å^2^ (approximately 13–16%), while Leu-115 and Thr-116 contributed 76.52–90.78 Å^2^ (approximately 5.6–6.8%) and 5.76–50.58 Å^2^ (approximately 0.4–3.7%), respectively. Indeed, our functional experiments confirm the critical roles of Glu-114 in enhancing the binding affinity of HP23L and its inhibitory potency on HIV-1-mediated cell fusion and entry, providing a linkage for the structural information and functional data.

Emerging studies suggest that lipopeptide-based fusion inhibitors have greatly improved antiviral activity and extended *in vivo* half-life, which are considered to anchor preferentially to the cell membrane where viral fusion occurs thus concentrating the inhibitors at the target site (Wexler-Cohen and Shai, 2007, 2009; Ingallinella et al., 2009; Augusto et al., 2014; Pessi, 2015; Chong et al., 2016, 2017). The lipopeptide LP-11 generated from adding fatty acid to the C-terminus of HP23L displayed extremely enhanced α-helicity, thermal stability and extended half-life, and it was physically stable in store of high temperature and humidity (Chong et al., 2016). Although we failed to build the conformation of the fatty acid molecule in the structure owing to its low electronic density, the present data demonstrated that the lipid conjugation did not significantly affect the binding networks of inhibitors. In comparison, the structures of HP23L and LP-11 had the same salt-bridges, hydrogen bonds and the similar hydrophobic interactions, together critically determining the stability of the 6-HBs. This finding also suggested that the increased α-helicity and thermostability of LP-11 might be due to its intrinsic properties as a lipopeptide, such as the oligomeric status. Indeed, our studies showed that the isolated HP23L self-assembled into a trimer while LP-11 existed as a hexamer in solution (data not shown). It is generally accepted that the physical stability of a peptide or protein would benefit from a high-order oligomeric structure. Recently, Figueira et al. reported that lipopeptide fusion inhibitors against measles virus self-assembled into nanoparticles until reaching the target cells, and it was thought that the self-assembly feature enhanced bio-distribution and half-life of the peptides while integration into the target cell membrane increased the inhibitory potency (Figueira et al., 2017).

We previously demonstrated that the M-T hook structure could confer fusion inhibitors with a high genetic barrier to inducing resistance, such as MT-C34, MT-SFT, MT-SC22EK, and MT-SC29EK (Chong et al., 2014a,b). As short-peptide inhibitors composed of the M-T hook structure and the pocket-binding domain, HP23, HP23L, and its lipopeptide derivative LP-11 specifically target the highly conserved pocket site of gp41, thus it is conceivable that they possess a higher genetic barrier for resistance. Indeed, we have so far failed to select HIV-1 variants resistant to HP23, HP23L, and LP-11 despite of considerable efforts. However, we previously obtained a panel of HIV-1 variants resistant to their template peptides (SC22EK and MT-SC22EK) during the *in vitro* selection, and the E49K and L57R mutations were identified as conferring cross-resistance to the newly-designed short-peptide inhibitors, including HP23L and LP-11 (Su et al., 2015a,b; Chong et al., 2016). Here, the crystal structures of HP23L bound to a wild-type or mutant target peptide have provided detail information to understand the molecular mechanism of HIV-1 resistance. Briefly, E49K substitution disrupted the intrahelical salt-bridge with His-53 and afterwards disturbed the interhelical hydrogen bond between the His-53 and Tyr-127 on inhibitors destroying the stability of α-helical and binding force with inhibitors. Moreover, the E49K mutation increased the positively charged repulsion around the mutation site accompanied by the positively charged residues nearby, such as anteroposterior His-53, Arg-46 and Lys-136 at C-terminal of HP23L, eventually resulting in instability of the 6-HB. As analyzed in our previous studies, the L57R mutation rudely disrupted its huge interactions with the inhibitors, including its hydrophobic interactions with the key hook structure-forming residue (Leu-115), the pocket-binding residues (Leu-115, Trp-120, Ile-124), and the residue located in the middle of inhibitor (Tyr-127). The L57R mutation also disturbed the direction of the side chains of Glu-118 thus severely destroying the hydrogen bond of the N-terminus of the inhibitors; and more, the positively charged Arg-57 also had repulsion force with the positively charged Lys-123 on the inhibitors. Together, these changes dramatically decreased the binding force of inhibitors, thereby critically determining viral resistance to the short-peptide fusion inhibitors targeting the deep pocket. It will be interesting to develop new peptides that can overcome the E49K and L57R-mediated resistance by applying different design strategies, such as engineering the peptide sequence based on the gp41 tripartite model and an artificial tail anchor (Su et al., 2017a,b).

In summary, we have obtained three sets of crystal structures for the 6-HBs formed by HP23L and LP-11 with the target-mimic peptides. The present structures have offered important information for understanding the structure and function of HIV-1 fusion inhibitors and the mechanism of viral resistance to short-peptide inhibitors that specifically target the gp41 pocket site. Definitely, the new data will guide to design more active fusion inhibitors against both wild-type and drug-resistant HIV-1 isolates.

## Author contributions

JH, XW, and YH: Designed the study and wrote the paper; XZ, YZ, HH, SZ, PW, and HC: Performed the experiments and analyzed the data. All authors reviewed the results and approved the final version of the manuscript.

### Conflict of interest statement

The authors declare that the research was conducted in the absence of any commercial or financial relationships that could be construed as a potential conflict of interest.

## References

[B1] Adams P. D. Grosse-Kunstleve R. W. Hung L. W. Ioerger T. R. McCoy A. J. Moriarty N. W.. (2002). PHENIX: building new software for automated crystallographic structure determination. Acta Crystallogr. D Biol. Crystallogr. 58(Pt 11), 1948–1954. 10.1107/S0907444902016657 12393927

[B2] Ashkenazi A. Wexler-Cohen Y. Shai Y. (2011). Multifaceted action of Fuzeon as virus-cell membrane fusion inhibitor. Biochim. Biophys. Acta 1808, 2352–2358. 10.1016/j.020 21762676

[B3] Augusto M. T. Hollmann A. Castanho M. A. Porotto M. Pessi A. Santos N. C. (2014). Improvement of HIV fusion inhibitor C34 efficacy by membrane anchoring and enhanced exposure. J. Antimicrob. Chemother. 69, 1286–1297. 10.1093/jac/dkt529 24464268 PMC3977611

[B4] Baldwin C. E. Sanders R. W. Deng Y. Jurriaans S. Lange J. M. Lu M.. (2004). Emergence of a drug-dependent human immunodeficiency virus type 1 variant during therapy with the T20 fusion inhibitor. J. Virol. 78, 12428–12437. 10.1128/JVI.78.22.12428-12437.2004 15507629 PMC525057

[B5] Berkhout B. Eggink D. Sanders R. W. (2012). Is there a future for antiviral fusion inhibitors? Curr. Opin. Virol. 2, 50–59. 10.1016/j.coviro.2012.01.002 22440966

[B6] Berkhout B. Sanders R. W. (2011). Molecular strategies to design an escape-proof antiviral therapy. Antiviral Res. 92, 7–14. 10.1016/j.antiviral.2011.04.002 21513746

[B7] Chan D. C. Chutkowski C. T. Kim P. S. (1998). Evidence that a prominent cavity in the coiled coil of HIV type 1 gp41 is an attractive drug target. Proc. Natl. Acad. Sci. U.S.A. 95, 15613–15617. 10.1073/pnas.95.26.15613 9861018 PMC28092

[B8] Chan D. C. Fass D. Berger J. M. Kim P. S. (1997). Core structure of gp41 from the HIV envelope glycoprotein. Cell 89, 263–273. 10.1016/S0092-8674(00)80205-6 9108481

[B9] Chan D. C. Kim P. S. (1998). HIV entry and its inhibition. Cell 93, 681–684. 10.1016/S0092-8674(00)81430-0 9630213

[B10] Chinnadurai R. Rajan D. Munch J. Kirchhoff F. (2007). Human immunodeficiency virus type 1 variants resistant to first- and second-version fusion inhibitors and cytopathic in *ex vivo* human lymphoid tissue. J. Virol. 81, 6563–6572. 10.1128/JVI.02546-06 17428857 PMC1900115

[B11] Chong H. Qiu Z. Sun J. Qiao Y. Li X. He Y. (2014a). Two M-T hook residues greatly improve the antiviral activity and resistance profile of the HIV-1 fusion inhibitor SC29EK. Retrovirology 11:40. 10.1186/1742-4690-11-40 24884671 PMC4046051

[B12] Chong H. Qiu Z. Su Y. He Y. (2015a). The N-terminal T-T motif of a third-generation HIV-1 fusion inhibitor is not required for binding affinity and antiviral activity. J. Med. Chem. 58, 6378–6388. 10.1021/acs.jmedchem.5b00109 26256053

[B13] Chong H. Qiu Z. Su Y. Yang L. He Y. (2015b). Design of a highly potent HIV-1 fusion inhibitor targeting the gp41 pocket. AIDS 29, 13–21. 10.1097/QAD.0000000000000498 25562490

[B14] Chong H. Wu X. Su Y. He Y. (2016). Development of potent and long-acting HIV-1 fusion inhibitors. AIDS 30, 1187–1196. 10.1097/QAD.0000000000001073 26919736

[B15] Chong H. Xue J. Xiong S. Cong Z. Ding X. Zhu Y.. (2017). A lipopeptide HIV-1/2 fusion inhibitor with highly potent *in vitro, ex vivo* and *in vivo* antiviral activity. J. Virol. 91:e00288-17. 10.1128/JVI.00288-17 28356533 PMC5432875

[B16] Chong H. Yao X. Qiu Z. Sun J. Qiao Y. Zhang M.. (2014b). The M-T hook structure increases the potency of HIV-1 fusion inhibitor sifuvirtide and overcomes drug resistance. J. Antimicrob. Chemother. 69, 2759–2769. 10.1093/jac/dku183 24908047

[B17] Chong H. Yao X. Qiu Z. Sun J. Zhang M. Waltersperger S.. (2013). Short-peptide fusion inhibitors with high potency against wild-type and enfuvirtide-resistant HIV-1. FASEB J. 27, 1203–1213. 10.1096/fj.12-222547 23233535

[B18] Chong H. Yao X. Sun J. Qiu Z. Zhang M. Waltersperger S.. (2012a). The M-T hook structure is critical for design of HIV-1 fusion inhibitors. J. Biol. Chem. 287, 34558–34568. 10.1074/jbc.M112.390393 22879603 PMC3464562

[B19] Chong H. Yao X. Zhang C. Cai L. Cui S. Wang Y.. (2012b). Biophysical property and broad anti-HIV activity of albuvirtide, a 3-maleimimidopropionic acid-modified peptide fusion inhibitor. PLoS ONE 7:e32599. 10.1371/journal.pone.0032599 22403678 PMC3293837

[B20] Colman P. M. Lawrence M. C. (2003). The structural biology of type I viral membrane fusion. Nat. Rev. Mol. Cell Biol. 4, 309–319. 10.1038/nrm1076 12671653

[B21] Dwyer J. J. Wilson K. L. Davison D. K. Freel S. A. Seedorff J. E. Wring S. A.. (2007). Design of helical, oligomeric HIV-1 fusion inhibitor peptides with potent activity against enfuvirtide-resistant virus. Proc. Natl. Acad. Sci. U.S.A. 104, 12772–12777. 10.1073/pnas.0701478104 17640899 PMC1937542

[B22] Eckert D. M. Kim P. S. (2001). Mechanisms of viral membrane fusion and its inhibition. Annu. Rev. Biochem. 70, 777–810. 10.1146/annurev.biochem.70.1.777 11395423

[B23] Eckert D. M. Malashkevich V. N. Hong L. H. Carr P. A. Kim P. S. (1999). Inhibiting HIV-1 entry: discovery of D-peptide inhibitors that target the gp41 coiled-coil pocket. Cell 99, 103–115. 10.1016/S0092-8674(00)80066-5 10520998

[B24] Eggink D. Baldwin C. E. Deng Y. Langedijk J. P. Lu M. Sanders R. W.. (2008). Selection of T1249-resistant human immunodeficiency virus type 1 variants. J. Virol. 82, 6678–6688. 10.1128/JVI.00352-08 18434391 PMC2447091

[B25] Eggink D. Berkhout B. Sanders R. W. (2010). Inhibition of HIV-1 by fusion inhibitors. Curr. Pharm. Des. 16, 3716–3728. 10.2174/138161210794079218 21128887

[B26] Eggink D. Bontjer I. Langedijk J. P. Berkhout B. Sanders R. W. (2011). Resistance of human immunodeficiency virus type 1 to a third-generation fusion inhibitor requires multiple mutations in gp41 and is accompanied by a dramatic loss of gp41 function. J. Virol. 85, 10785–10797. 10.1128/JVI.05331-11 21835789 PMC3187517

[B27] Eggink D. Langedijk J. P. Bonvin A. M. Deng Y. Lu M. Berkhout B.. (2009). Detailed mechanistic insights into HIV-1 sensitivity to three generations of fusion inhibitors. J. Biol. Chem. 284, 26941–26950. 10.1074/jbc.M109.004416 19617355 PMC2785381

[B28] Emsley P. Cowtan K. (2004). Coot: model-building tools for molecular graphics. Acta Crystallogr. D Biol. Crystallogr. 60(12 Pt 1), 2126–2132. 10.1107/S0907444904019158 15572765

[B29] Figueira T. N. Palermo L. M. Veiga A. S. Huey D. Alabi C. A. Santos N. C.. (2017). *In vivo* efficacy of measles virus fusion protein-derived peptides is modulated by the properties of self-assembly and membrane residence. J. Virol. 91:e01554-16. 10.1128/JVI.01554-16 27733647 PMC5165226

[B30] Greenberg M. L. Cammack N. (2004). Resistance to enfuvirtide, the first HIV fusion inhibitor. J. Antimicrob. Chemother. 54, 333–340. 10.1093/jac/dkh330 15231762

[B31] He Y. (2013). Synthesized peptide inhibitors of HIV-1 gp41-dependent membrane fusion. Curr. Pharm. Des. 19, 1800–1809. 10.2174/1381612811319100004 23092277

[B32] He Y. Cheng J. Li J. Qi Z. Lu H. Dong M.. (2008a). Identification of a critical motif for the human immunodeficiency virus type 1 (HIV-1) gp41 core structure: implications for designing novel anti-HIV fusion inhibitors. J. Virol. 82, 6349–6358. 10.1128/JVI.00319-08 18417584 PMC2447044

[B33] He Y. Cheng J. Lu H. Li J. Hu J. Qi Z.. (2008b). Potent HIV fusion inhibitors against Enfuvirtide-resistant HIV-1 strains. Proc. Natl. Acad. Sci. U.S.A. 105, 16332–16337. 10.1073/pnas.0807335105 18852475 PMC2571013

[B34] He Y. Xiao Y. Song H. Liang Q. Ju D. Chen X.. (2008c). Design and evaluation of sifuvirtide, a novel HIV-1 fusion inhibitor. J. Biol. Chem. 283, 11126–11134. 10.1074/jbc.M800200200 18303020

[B35] Heil M. L. Decker J. M. Sfakianos J. N. Shaw G. M. Hunter E. Derdeyn C. A. (2004). Determinants of human immunodeficiency virus type 1 baseline susceptibility to the fusion inhibitors enfuvirtide and T-649 reside outside the peptide interaction site. J. Virol. 78, 7582–7589. 10.1128/JVI.78.14.7582-7589.2004 15220433 PMC434069

[B36] Ingallinella P. Bianchi E. Ladwa N. A. Wang Y. J. Hrin R. Veneziano M.. (2009). Addition of a cholesterol group to an HIV-1 peptide fusion inhibitor dramatically increases its antiviral potency. Proc. Natl. Acad. Sci. U.S.A. 106, 5801–5806. 10.1073/pnas.0901007106 19297617 PMC2667053

[B37] Ishikawa H. Meng F. Kondo N. Iwamoto A. Matsuda Z. (2012). Generation of a dual-functional split-reporter protein for monitoring membrane fusion using self-associating split GFP. Protein Eng. Des. Sel. 25, 813–820. 10.1093/protein/gzs051 22942393

[B38] Jiang S. Lu H. Liu S. Zhao Q. He Y. Debnath A. K. (2004). N-substituted pyrrole derivatives as novel human immunodeficiency virus type 1 entry inhibitors that interfere with the gp41 six-helix bundle formation and block virus fusion. Antimicrob. Agents Chemother. 48, 4349–4359. 10.1128/AAC.48.11.4349-4359.2004 15504864 PMC525433

[B39] Kilby J. M. Hopkins S. Venetta T. M. DiMassimo B. Cloud G. A. Lee J. Y.. (1998). Potent suppression of HIV-1 replication in humans by T-20, a peptide inhibitor of gp41-mediated virus entry. Nat. Med. 4, 1302–1307. 10.1038/3293 9809555

[B40] Lalezari J. P. Henry K. O'Hearn M. Montaner J. S. Piliero P. J. Trottier B.. (2003). Enfuvirtide, an HIV-1 fusion inhibitor, for drug-resistant HIV infection in North and South America. N. Engl. J. Med. 348, 2175–2185. 10.1056/NEJMoa035026 12637625

[B41] Laskowski R. A. MacArthur M. W. Moss D. S. Thornton J. M. (1993). PROCHECK: a program to check the stereochemical quality of protein structures. J. Appl. Crystallogr. 26, 283–291. 10.1107/S0021889892009944

[B42] Liu Z. Shan M. Li L. Lu L. Meng S. Chen C.. (2011). *In vitro* selection and characterization of HIV-1 variants with increased resistance to sifuvirtide, a novel HIV-1 fusion inhibitor. J. Biol. Chem. 286, 3277–3287. 10.1074/jbc.M110.199323 21098485 PMC3030333

[B43] Lobritz M. A. Ratcliff A. N. Arts E. J. (2010). HIV-1 Entry, inhibitors, and resistance. Viruses 2, 1069–1105. 10.3390/v2051069 21994672 PMC3187606

[B44] Matthews T. Salgo M. Greenberg M. Chung J. DeMasi R. Bolognesi D. (2004). Enfuvirtide: the first therapy to inhibit the entry of HIV-1 into host CD4 lymphocytes. Nat. Rev. Drug Discov. 3, 215–225. 10.1038/nrd1331 15031735

[B45] McCoy A. J. Grosse-Kunstleve R. W. Adams P. D. Winn M. D. Storoni L. C. Read R. J. (2007). Phaser crystallographic software. J. Appl. Crystallogr. 40(Pt 4), 658–674. 10.1107/S0021889807021206 19461840 PMC2483472

[B46] Naito T. Izumi K. Kodama E. Sakagami Y. Kajiwara K. Nishikawa H.. (2009). SC29EK, a peptide fusion inhibitor with enhanced alpha-helicity, inhibits replication of human immunodeficiency virus type 1 mutants resistant to enfuvirtide. Antimicrob. Agents Chemother. 53, 1013–1018. 10.1128/AAC.01211-08 19114674 PMC2650564

[B47] Nameki D. Kodama E. Ikeuchi M. Mabuchi N. Otaka A. Tamamura H.. (2005). Mutations conferring resistance to human immunodeficiency virus type 1 fusion inhibitors are restricted by gp41 and Rev-responsive element functions. J. Virol. 79, 764–770. 10.1128/JVI.79.2.764-770.2005 15613304 PMC538534

[B48] Otaka A. Nakamura M. Nameki D. Kodama E. Uchiyama S. Nakamura S.. (2002). Remodeling of gp41-C34 peptide leads to highly effective inhibitors of the fusion of HIV-1 with target cells. Angew. Chem. Int. Ed. Engl. 41, 2937–2940. 10.1002/1521-3773(20020816)41:16<2937::AID-ANIE2937>3.0.CO;2-J 12203417

[B49] Otwinowski Z. Minor W. (1997). Processing of X-ray diffraction data collected in oscillation mode. Meth. Enzymol. 276, 307–326. 10.1016/S0076-6879(97)76066-X 27754618

[B50] Pessi A. (2015). Cholesterol-conjugated peptide antivirals: a path to a rapid response to emerging viral diseases. J. Pept. Sci. 21, 379–386. 10.1002/psc.2706 25331523 PMC7167725

[B51] Rimsky L. T. Shugars D. C. Matthews T. J. (1998). Determinants of human immunodeficiency virus type 1 resistance to gp41-derived inhibitory peptides. J. Virol. 72, 986–993. 9444991 10.1128/jvi.72.2.986-993.1998 PMC124569

[B52] Shimura K. Nameki D. Kajiwara K. Watanabe K. Sakagami Y. Oishi S.. (2010). Resistance profiles of novel electrostatically constrained HIV-1 fusion inhibitors. J. Biol. Chem. 285, 39471–39480. 10.1074/jbc.M110.145789 20937812 PMC2998136

[B53] Sista P. R. Melby T. Davison D. Jin L. Mosier S. Mink M.. (2004). Characterization of determinants of genotypic and phenotypic resistance to enfuvirtide in baseline and on-treatment HIV-1 isolates. AIDS 18, 1787–1794. 10.1097/00002030-200409030-00007 15316339

[B54] Steffen I. Pöhlmann S. (2010). Peptide-based inhibitors of the HIV envelope protein and other class I viral fusion proteins. Curr. Pharm. Des. 16, 1143–1158. 10.2174/138161210790963751 20030613

[B55] Su S. Wang Q. Xu W. Yu F. Hua C. Zhu Y.. (2017a). A novel HIV-1 gp41 tripartite model for rational design of HIV-1 fusion inhibitors with improved antiviral activity. AIDS 31, 885–894. 10.1097/QAD.0000000000001415 28121713

[B56] Su S. Zhu Y. Ye S. Qi Q. Xia S. Ma Z.. (2017b). Creating an Artificial tail anchor as a novel strategy to enhance the potency of peptide-based HIV fusion inhibitors. J. Virol. 91:e01445-16. 10.1128/JVI.01445-16 27795416 PMC5165219

[B57] Su Y. Chong H. Qiu Z. Xiong S. He Y. (2015a). Mechanism of HIV-1 resistance to short-peptide fusion inhibitors targeting the Gp41 pocket. J. Virol. 89, 5801–5811. 10.1128/JVI.00373-15 25787278 PMC4442445

[B58] Su Y. Chong H. Xiong S. Qiao Y. Qiu Z. He Y. (2015b). Genetic pathway of HIV-1 resistance to novel fusion inhibitors targeting the Gp41 pocket. J. Virol. 89, 12467–12479. 10.1128/JVI.01741-15 26446597 PMC4665254

[B59] Tan K. Liu J. Wang J. Shen S. Lu M. (1997). Atomic structure of a thermostable subdomain of HIV-1 gp41. Proc. Natl. Acad. Sci. U.S.A. 94, 12303–12308. 10.1073/pnas.94.23.12303 9356444 PMC24915

[B60] Weissenhorn W. Dessen A. Harrison S. C. Skehel J. J. Wiley D. C. (1997). Atomic structure of the ectodomain from HIV-1 gp41. Nature 387, 426–430. 10.1038/387426a0 9163431

[B61] Welch B. D. VanDemark A. P. Heroux A. Hill C. P. Kay M. S. (2007). Potent D-peptide inhibitors of HIV-1 entry. Proc. Natl. Acad. Sci. U.S.A. 104, 16828–16833. 10.1073/pnas.0708109104 17942675 PMC2040420

[B62] Wexler-Cohen Y. Shai Y. (2007). Demonstrating the C-terminal boundary of the HIV 1 fusion conformation in a dynamic ongoing fusion process and implication for fusion inhibition. FASEB J. 21, 3677–3684. 10.1096/fj.07-8582com 17575260

[B63] Wexler-Cohen Y. Shai Y. (2009). Membrane-anchored HIV-1 N-heptad repeat peptides are highly potent cell fusion inhibitors via an altered mode of action. PLoS Pathog. 5:e1000509. 10.1371/journal.ppat.1000509 19593361 PMC2699469

[B64] Xiong S. Borrego P. Ding X. Zhu Y. Martins A. Chong H.. (2017). A helical short-peptide fusion inhibitor with highly potent activity against Human Immunodeficiency Virus Type 1 (HIV-1), HIV-2, and simian immunodeficiency virus. J. Virol. 91:e01839-16. 10.1128/JVI.01839-16 27795437 PMC5165200

[B65] Xu L. Pozniak A. Wildfire A. Stanfield-Oakley S. A. Mosier S. M. Ratcliffe D.. (2005). Emergence and evolution of enfuvirtide resistance following long-term therapy involves heptad repeat 2 mutations within gp41. Antimicrob. Agents Chemother. 49, 1113–1119. 10.1128/AAC.49.3.1113-1119.2005 15728911 PMC549241

[B66] Yu F. Lu L. Liu Q. Yu X. Wang L. He E.. (2014). ADS-J1 inhibits HIV-1 infection and membrane fusion by targeting the highly conserved pocket in the gp41 NHR-trimer. Biochim. Biophys. Acta 1838, 1296–1305. 10.1016/j.bbamem.2013.12.022 24388952

